# Survey of Management Practices and Farmers’ Perceptions of Diseases on Organic Dairy Cattle Farms in California

**DOI:** 10.3390/ani12192526

**Published:** 2022-09-21

**Authors:** Sejin Cheong, Juliette Di Francesco, Kyuyoung Lee, Richard Van Vleck Pereira, Randi Black, Betsy Karle, Melissa Lema, Alda F. A. Pires

**Affiliations:** 1 Department of Population Health and Reproduction, School of Veterinary Medicine, University of California-Davis, Davis, CA 95616, USA; 2 Center for Animal Disease Modeling and Surveillance (CADMS), Department of Medicine and Epidemiology, School of Veterinary Medicine, University of California-Davis, Davis, CA 95616, USA; 3 Cooperative Extension Sonoma County, Division of Agriculture and Natural Resources, University of California, Santa Rosa, CA 95403, USA; 4 Cooperative Extension Glenn County, Division of Agriculture and Natural Resources, University of California, Orland, CA 95963, USA; 5 North Coast and Organic Field Service, Western United Dairies, Turlock, CA 95380, USA

**Keywords:** dairy cattle, California, organic

## Abstract

**Simple Summary:**

In 2019, California accounted for approximately 40% of organic products in the US, and dairy products and milk were the top organic commodity in the state. The objective of this study was to describe organic dairy cattle farmers’ management practices and perceptions of diseases in California. A questionnaire on farm history and demographics, animal diseases, parasite problems, housing and pasture management, and organic education, was mailed to 160 organic dairy farms, of which 36 responded. Respondents were more likely to report mastitis in cows, pinkeye in heifers, and digestive problems in calves, as issues affecting their stock “often” or “almost always” in the last 12 months. Although most farmers vaccinated their cattle against Bovine Viral Diarrhea and *Escherichia coli* mastitis, they still described that these diseases frequently impacted their animals. Over half of the farmers did not perceive gastrointestinal parasites or biting flies to be a problem and did not observe signs of lice and mites. According to the results, the management of disease in all age classes is a concern; options and efficacies of alternative therapeutic methods, as well as preventive measures for organic dairies need to be further explored.

**Abstract:**

In 2019, California accounted for approximately 40% of organic products in the US, and dairy products and milk were the top organic commodity in the state. The objective of this study was to describe organic dairy cattle farmers’ management practices and perceptions of diseases in California. A survey inquiring about farm history and demographics, animal diseases, parasite problems, housing and pasture management, and organic education, was mailed to 160 organic dairy farms, of which 36 (22.5%) responded. Among respondents, the majority (83.9%) were located in Northern California; median farm size was 310 cows, and the dominant breed was Holstein (60.0%). Respondents were more likely to report mastitis in cows (45.2%), pinkeye in heifers (31.3%), and digestive problems in calves (47.0%), as issues affecting their stock “often” or “almost always” in the last 12 months. Although most farmers vaccinated their cattle against Bovine Viral Diarrhea (86.1%) and *Escherichia coli* mastitis (80.6%), they still described that these diseases frequently impacted their animals. Over half of the farmers did not perceive gastrointestinal parasites or biting flies to be a problem and did not observe signs of lice and mites. According to the results, the management of disease in all age classes is a concern; options and efficacies of alternative therapeutic methods, as well as preventive measures for organic dairies need to be further explored.

## 1. Introduction

The production of certified organic commodities has significantly increased over the past few years in the United States (US). The survey of organic farms by the US Department of Agriculture (USDA) in 2019 reported that sales of organic commodities had increased by around 30% since 2016 [1,2]. In 2019, California (CA) produced approximately 40% of organic products in the US. The value of Californian organic commodities represented more than $10.4 billion, and dairy products and milk were the primary organic commodity ($7.4 billion) [3]. 

According to a survey of management practices on organic and conventional farms carried out in Minnesota (MN), the reasons for producers to carry out organic farming were: minimizing the use of pesticides and the potential health issues caused by their use; economic benefits; and acknowledging the advantages for the soil and environment [4]. Another survey of members of the North Carolina State University Sensory Service Center database found that many consumers were highly motivated to purchase organic milk because of concerns over their own health, the ethical treatment of animals, and support of local farmers [5]. In studies investigating the characteristics of organic milk consumers [6,7], the major reasons paying for organic milk were the perceived quality of organic milk and the eco-friendliness of being a primary consumer.

The National Organic Program (NOP) of the USDA develops the rules and regulations for organic products and livestock in the US [8]. The regulation requires that livestock operations only feed and use organic agricultural products (e.g., pasture, forages, and plant materials used for bedding). Specifically, ruminants are required to get on average at least 30% of dry matter intake from pasture during the grazing season, and to have access to pasture a minimum of 120 days per year. Additionally, organic farmers are prohibited from administering any antibiotics and parasiticides (except for fenbendazole and moxidectin that are approved for restricted, non-routine use), or hormones (except for oxytocin during post-parturition care) to their livestock, in the absence of illness. Antibiotic or parasiticide use is only permitted in emergency care under veterinary oversight. Indeed, regulation does not allow producers to withhold the adequate treatment of sick or injured animals to maintain their organic status. If animals are treated with medications that are not authorized in organic production (e.g., antibiotics), they lose their organic status and have to be sold on a non-organic market. This differs from European and Canadian regulations that allow the use of antibiotics or parasiticides with extended withdrawal times, without the loss of organic status [9,10]. Consequently, substantial limitations in the medications that can be used in organic farms in the US increase the challenges for treatment options and emphasize the importance of preventive management practices and adequate health care, such as administering vaccines and organic dietary supplements or additives [11].

Previous studies, conducted in the Midwest US, compared management practices between organic and conventional farms, including housing and water sources, antibiotic use, and parasite management practices [4,12,13,14]. The organic farms described in these studies shared some specific features, such as a high percentage of using rotational grazing in pastures [12], and providing natural feed additives (e.g., kelp or seaweed) to avoid using chemicals agents (e.g., ionophores) [4]. Another study in the Midwest US reported that certain management practices adopted by organic farms could reduce disease spread, such as housing pre-weaned calves in an individual area and using automatic individual water bowls for adult cows, in contrast to sharing the water source in conventional farms [13]. 

Previous research in CA has characterized dairy health management in both conventional and organic dairy cattle farms, but did not detail the specificities of organic farms [15,16]. Consequently, little is known regarding general husbandry practices, or common diseases on organic dairy cattle farms in CA. Our study aimed to fill these knowledge gaps by describing management practices and perceptions of diseases by farmers in organic dairy cattle farms in CA.

## 2. Materials and Methods

### 2.1. Participant Recruitment and Survey Administration

The present cross-sectional study recruited participants from the list of organic dairy cattle farms in CA generated from a publicly available list on the USDA website, and through a list provided by the CA Department of Food and Agriculture (CDFA). First, we created an initial list of organic farms using several keywords (i.e., “livestock”, “dairy”, “milk”, and “cow”) from both USDA and CDFA lists, with the goal of ensuring that the farms in these lists were dairy producers. Secondly, we only included organic dairy farms certified organic “Grade A” with the USDA NOP; the reason for this was that “Grade A” milk (also known as fluid grade milk) guaranteed that the farmer follows FDA regulation, defining minimum sanitation standards expected for all farms included in the study [17]. After this selection criteria process and the removal of duplicates, a total of 160 organic dairy farms were included in the survey.

A mixed-mode survey approach (i.e., both mailed hardcopy and access to the web-based survey) was conducted with multiple mailings to maximize response rates [18]. The introductory postcards and survey packets included a cover letter with general information about the survey, a copy of the questionnaire, and a pre-stamped envelope for the return of the survey. The survey packet was first mailed in November 2018. A follow-up e-mail was sent two weeks later, and a final postcard reminder was mailed in March 2019. The final postcards included the weblink and the QR code for the survey, providing access to the online survey to farmers. The survey was available from November 2018 to July 2019. As an incentive for participating, farmers who completed and returned the survey were sent a gift card by post.

The survey protocol of this study was reviewed and approved by the Institutional Review Board Administration at the University of California, Davis (#1303548-1). The questionnaire was pre-tested by the research team with three farmers and several industry representatives before it was mailed to farms.

### 2.2. Survey Questionnaires

The survey consisted of 45 questions divided into six different sections (File S1):

(i) Respondent information. The first section included questions about general farm background information (e.g., how long the farm had been operating and certified organic). 

(ii) Farm demographics. The second section focused on the properties of the dairy farm including predominant dairy breed; average milk production; average bulk tank somatic cell count (BTSCC); number of dairy animals by age class (i.e., cows, heifers, calves); and the reasons for which dairy animals were removed/culled or died/were euthanized during the past 12 months. 

(iii) Animal disease management. The third section addressed the frequencies of diseases/disorders that most impacted dairy animals as perceived by the farmers; the medications or supplements and vaccines used in the farm; and the frequency of routine veterinarians’ visits and services provided by them. 

(iv) Parasite-specific questions. The fourth section focused on the perceptions of the farmers regarding parasite problems, including gastrointestinal parasites, lice and mites, and biting flies, as well as the use of de-wormers. 

(v) Housing and pasture management. The fifth section addressed grazing schedule; pasture management practices; and heat abatement methods. 

(vi) Organic education and outreach. The last section focused on how the respondents preferred to receive information on dairy health and how frequently they sought out such information.

### 2.3. Data Management and Statistical Analysis

The questionnaire included binary (yes/no), categorical (multiple choice), and short-open-ended questions. Some categorical questions allowed the respondents to select multiple answers (e.g., vaccine administration and the primary sources of information for disease treatment and management options). Short-open-ended questions included: the name of the county where the farm was located; the average milk production per cow per day; the number of animals by age class (i.e., cows, heifers, calves); the number of acres of designated organic pastures; and the reasons why antibiotics were administered for the respondents who answered ‘yes’ to whether they have used antibiotics in past 12 months. 

The responses delivered via mail were entered manually into an excel sheet and aggregated with the online-collected data. All the analyses were performed in R (version 3.6.1). Descriptive statistics were used to summarize the survey results. Categorical variables were summarized as counts, proportions, and 95% confidence intervals (CI), and these were calculated using only the number of participants who responded to that specific question (i.e., for all questions, unanswered responses were considered as missing data). For the question on medications or supplements used, vitamin A-D-E in feed and vitamin A-D-E injection were recategorized as one, as were selenium in feed and selenium injection, since we focused on the use of medications or supplements and not on their administration methods. Continuous variable answers were summarized as median and range.

## 3. Results

**Figure 1:**
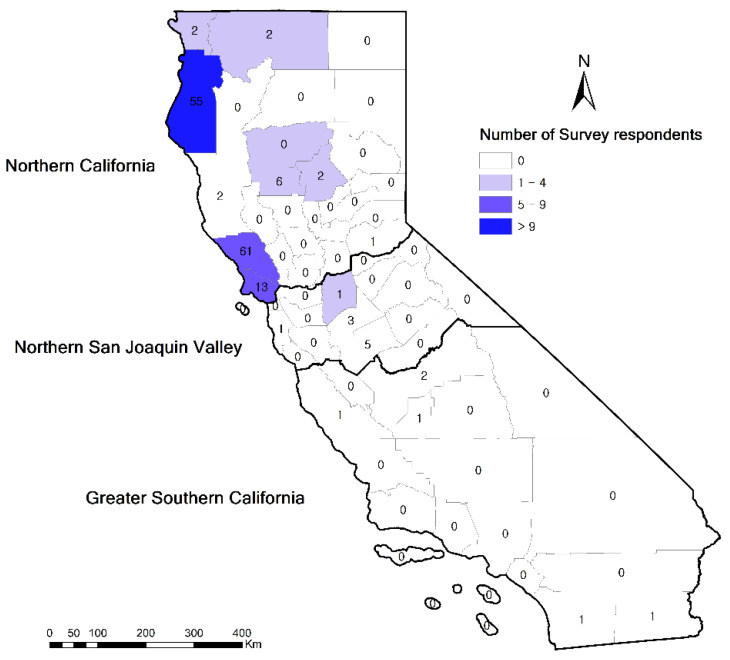
Map showing the number of organic farms and survey respondents in each county and the boundaries of the three CA regions. The numbers indicate the number of invited organic farms, and the colors indicate the number of survey respondents (n = 31 farms, 5 farms did not specify their county). This map was generated using ArcGIS (Environmental Systems Research Institute (ESRI), ArcGIS Release10.8.1, Redlands, CA, USA).

A total of 36 (22.5%) organic dairy farmers responded to the survey from a total of 160 invited participants: from these, 30 farmers responded by hard copies and six farmers responded through the online survey. The survey respondents were mostly the farm owners (80.5%, 29/36) and managers (16.7%, 6/36), and one survey was completed by an employee (2.8%, 1/36). All 36 farms were certified organic by USDA accredited certifiers, which included California Certified Organic Farmers (36.1%, 13/36), Marin Organic Certified Agriculture (22.2%, 8/36), Oregon Tilth Certified Organic (16.7%, 6/36), Organic Certifiers, Inc (19.4%,7/36), Quality Assurance International (2.8%, 1/36), and Global Culture (2.8%, 1/36). The median duration of organic farm accreditation was 12 years, ranging from 3 to 19 years. Farms were mainly located in Northern CA, with 32.3% (10/31) in Humboldt County, 29% (9/31) in Sonoma County, 19.4% (6/31) in Marin County, and 3.2% (1/31) in Del Norte, San Joaquin, Siskiyou, Butte, Glenn, and Tehama County, respectively (Figure 1). Based on the regional classification used in similar studies in CA [15,16,19,20], the response rate was highest in Northern CA (20.8%, 30/144), with only one response from Northern San Joaquin Valley (10.0%, 1/10), and no responses from Greater Southern CA (0.0%, 0/6) [16].

### 3.1. Farm Demographics

Demographics of the organic dairy cattle farms surveyed are summarized in Table 1. The median numbers of current dairy animals by age group were 310 (80–4000) cows, 170 (28–1000) heifers, and 86 (20–800) calves. Over the last 12 months, 29.4% (10/34) of farms had brought cows, heifers, calves, or bulls, into their operation. Newly introduced animals originated mostly from single farms (80.0%, 8/10) and none were brought in from club sales, dealers, or sale barns. The average milk production in participating farms was 25 kg/cow/day (15–36). Their average BTSCC of milk mostly ranged from 100,000 to 199,000 cells/mL (68.6%, 24/35) and no farms exceeded 300,000 cells/mL.

**Figure 2:**
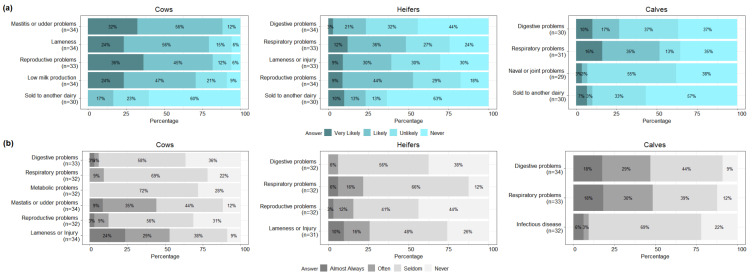
Reasons for permanent removal/culling (**a**) and for death/euthanasia (**b**) of dairy animals during the last 12 months by age class reported by farmers (n = 36 farms surveyed).

The reasons for the permanent removal/culling and death/euthanasia of dairy animals in the last 12 months are summarized by age class (Figure 2). The participants were most likely to report udder and mastitis problems (88.2%, 30/34) as the reason for the permanent removal/culling (answered as “likely” or “very likely”) of cows, while lameness or injury (52.9%, 18/34) were reported as the most likely causes of deaths/euthanasia (answered as “often” or “almost always”) in cows. For heifers, the participants were most likely to report reproductive problems (52.9%, 18/34) as the reason for the permanent removal/culling, and lameness or injury (25.8%, 8/31) as the cause of deaths/euthanasia. As for calves, participants reported that respiratory problems were the most likely reason for both removal/culling (51.6%, 16/31) and death/euthanasia (48.5%,16/33). The median of permanent removals/culls was 6, 1.5 times, and 4.3 times higher than the median of deaths/euthanasia in cows, heifers, and calves, respectively.

### 3.2. Animal Disease Management

**Figure 3:**
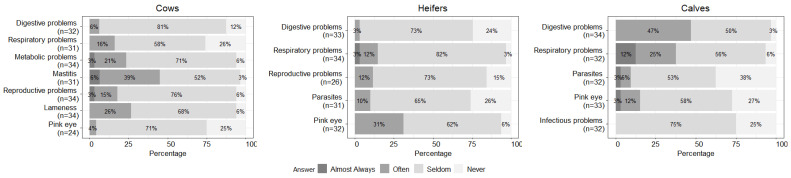
Frequency of diseases and disorders in each age class as reported by farmers (n = 36 farms surveyed).

Participants reported the frequencies of diseases and disorders that impacted their animals within each age class during the last 12 months (Figure 3). They indicated mastitis in cows (45.2%, 14/31), infectious bovine keratoconjunctivitis (IBK, pinkeye) in heifers (31.3%, 10/32), and digestive problems in calves (i.e., diarrhea, scours, and bloat; 47.0%, 16/34) as diseases and disorders “often” or “almost always” impacting their stock. 

**Figure 4:**
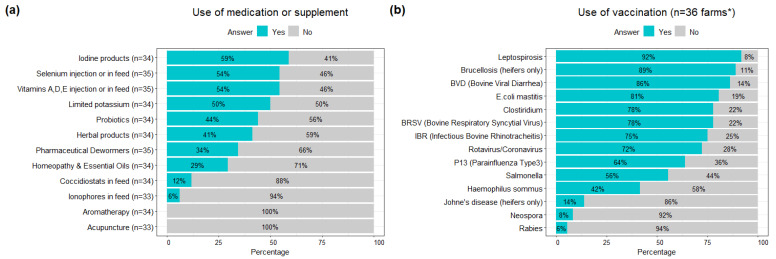
Use of medication or supplements (**a**) and vaccination (**b**) (n = 36 farms surveyed). * For the use of vaccination, respondents could select all that applied to them.

More than half of the farmers had administered antibiotics to their dairy cattle during the past 12 months at least once (52.8%, 19/36). Among the participants who specified the reasons for using antibiotics, the most common were treating respiratory diseases (47.0%, 8/17), mastitis (29.4%, 5/17), and lameness or foot rot (17.6%, 3/17). Most organic dairy farmers administered vaccines for key pathogens such as leptospirosis and brucellosis, and around half of the farmers generally used iodine products for the treatment of infections (Figure 4). 

The majority of respondents reported routine veterinarian visits (88.2%, 30/34), and their frequency was generally higher than once a month (43.3%, 13/30) or once every 1–3 months (43.3%, 13/30). During the visits, veterinarians mainly provided reproductive work (75.8%, 25/33), but treatments for sick cows (69.7%, 23/33) and drug prescriptions (63.6%, 21/33) also accounted for a high proportion of their services. However, respondents answered that the person who commonly identified and treated the sick cows was the herd manager (48.6%, 17/35 and 42.9%, 15/35, respectively) (Table S1).

### 3.3. Parasite-Specific Questions

More than half of the respondents did not consider gastrointestinal parasites (67.7%, 23/34) or biting flies (68.8%, 22/32) as problems, and indicated not observing signs of lice or mites, such as excessive scratching and rubbing, hair loss, or scabby skin conditions (75.0%, 24/32) (Table S2). Overall, only 6.2% of the respondents perceived both gastrointestinal parasites and biting flies as problems and had observed signs of lice or mites. When scheduling grazing, 61.8% (21/34) of the participating farms did not consider intestinal parasite prevention or control. Among the respondents who identified gastrointestinal parasites to be a problem (32.4%, 11/34), 63.6% (7/11) indicated that the most affected age group was heifers. Ivermectin was the most commonly used de-wormer (76.3%, 10/13). Diatomaceous earth or herbs were also frequently administered as a de-wormer (53.9%, 7/13). 

### 3.4. Housing and Pasture Management

The average surface of designated organic pasture was 413 acres (65–3500). The main method used for pasture management was rotational grazing (57.1%, 20/35), and the majority of the farms (91.4%, 32/35) did not let the cows and heifers graze on the same pasture as other livestock (Table 2). The farms which had other livestock grazing on the same pasture (8.6%, 3/35) were large (1,500, 1,800, and 3,500 acres, respectively). Free stalls were the most common type of housing used for lactating cows (71.4%, 25/35). Among the 15 farms grazing year-round, 40.0% (6/15) of the respondents considered intestinal parasite prevention/control when scheduling grazing.

### 3.5. Organic Education and Outreach

Most of the respondents preferred to receive general information through reading materials (e.g., magazines, newsletters, and fact sheets; 75.8%, 25/33), or obtaining information from peers and technical services personnel (e.g., farmers, farm advisors, veterinarians, company representatives, feed store employees; 72.7%, 24/33). Dairy health information, such as advice on disease prevention and treatment, was generally obtained from veterinarians (75.0%, 27/36), but nutritionists (38.9%, 14/36) and the internet (38.9%, 14/36) were also common sources of information. Fifty-four percent (19/35) of respondents sought out relevant information monthly, and 28.6% (10/35) weekly.

## 4. Discussion

The present survey investigated husbandry and management practices, as well as animal disease in organic dairy cattle farms in CA. Specifically, we described general demographic characteristics at the farm-level, farmers’ perceptions of diseases/disorders encountered by age class (i.e., cows, heifers, and calves), prevention approaches (i.e., supplementary medications and vaccines administered), perceptions of parasite problems, and common pasture and housing management practices. 

Most of the organic dairy cattle farms that participated in this study were located in Northern CA. Similarly, previous surveys on antimicrobial drug use and stewardship practices in CA had identified that most of the organic dairies were located in Northern CA, whereas approximately 80% of the conventional dairies were located in Northern San Joaquin Valley or Greater Southern CA [15,16]. Studies have shown that the geography of organic farms is associated with various environmental factors, including regional climates and landscape heterogeneity [21]; the difference in the distribution of conventional and organic farms across CA has previously been attributed to the milder climate of Northern CA, which is more favorable for pasture-based and organic farming, for which pasture availability is a key determining factor [19]. 

Several herd characteristics differed from other states, such as herd size, BTSCC, and breed. In this study, we found a median herd size of 310 cows (n = 36 farms), which is similar to the average herd sizes of 287 cows (n = 18 farms) and 381 cows recorded on organic farms in a previous CA survey, and in the West US by the USDA, respectively [15,22]. By contrast, the average size of organic herds documented in MN and Upper Midwest US by the USDA were 68 cows (n = 35 farms) and 64 cows, respectively [14]. The average BTSCC was lower in this study than in the MN survey [14]. According to a milk quality study carried out in Wisconsin (WI), a higher BTSCC is generally significantly associated with a smaller herd size [23], therefore, the smaller herd sizes of organic farms in MN may explain the higher BTSCC. Crossbreed cows were predominant in only 8.6% of farms in this study, and the majority of the cows (60.0%) were Holstein. In comparison, crossbreed cows accounted on average for 60% of the cattle on organic dairy farms in MN [4], while 27% of the organic farms in New York (NY), WI, and Oregon (OR), predominantly had crossbreed animals or cattle other than Holsteins and Jerseys [12]. This common preference of organic producers for crossbreeds has been linked to their superior fertility and longer survival [4,24]. 

In this study, mastitis was the most frequent disease reported in cows, which is similar to a recent study undertaken with organic dairy producers in Ohio (OH) [25]. Organic dairies seem to have fewer cases of clinical mastitis compared to conventional dairies [13,26], and this may depend on the disparity in the detection and reporting of cases between conventional and organic farms [11]. Since any usage of antimicrobial drugs leads to the loss of organic certification based on the NOP standards, alternative therapies (e.g., essential oils, probiotics, homeopathy, and herbal remedies) are generally the common treatment options for mastitis on organic farms [27]. However, the administration route and efficacy of alternative medications for treatment of clinical mastitis are controversial and need further assessment through experimental studies [25,28,29,30,31,32,33]. Currently, commercially available mastitis vaccines exist against *Escherichia coli* (*E. coli*)*, Staphylococcus aureus* (*S. aureus*)*,* and *Mycoplasma bovis* (*M. bovis*) [34]. Vaccines against coliform mastitis (*E. coli*) are commonly used on dairy operations in the Western US (35.7%), whereas those against *S. aureus* (0.3% in the Western US and 12.2% in CA) and *M. bovis* (0.0% in the Western US) are more rarely administered [15,34]. In this study, 80.6% of organic dairy participants vaccinated against *E. coli* mastitis, but the overall frequency of mastitis remained high. This could be linked to mastitis cases being caused by pathogens other than *E. coli*, or to insufficient preventative measures. Indeed, there was a high percentage of farms in the Western US which were positive for *S. aureus* (67.5%) and *Mycoplasma* (30.2%) via milk culture [35]. 

Pinkeye (IBK, caused by the bacterium *Moraxella bovis*) was the predominant disease in heifers. The reason for pinkeye being frequently observed in heifers and not in cows may be related to a higher vulnerability to infection of young animals [36], and to older animals having a higher level of natural immunity [37]. Several studies also indicate that pinkeye is associated with face flies [37,38,39,40], but few respondents (31.3%) perceived flies to be a problem in this study. Since there are currently no efficient vaccines against pinkeye [41], and the disease is typically treated with antibiotics such as oxytetracycline via subconjunctival or topical routes [38], the restriction in the use of antibiotics can be challenging for the control of this disease on organic farms. 

Digestive problems were the most frequently perceived disorder in calves. Various viruses and bacteria cause diarrhea in calves, but *E. coli*, bovine rotavirus, bovine coronavirus, and *Cryptosporidium,* are the main agents of diarrhea in calves aged 1 to 2 weeks [42,43]. Based on the risk factors assessed in other studies, the high frequency of diarrhea in calves reported in this study despite high vaccination rates against rotavirus, coronavirus, and bovine viral diarrhea (i.e., over 70–80%), may be due to housing management practices (e.g., absence of individual calving areas or housing), to the presence of respiratory diseases [44,45], or to a failure of passive immunity transfer [46]. Likewise, respiratory diseases were frequently reported in calves (37.5%) in this study. The efficacy of several preventive practices against bovine respiratory disease (BRD) through colostrum management and housing, as well as vaccination programs, have been discussed [47,48]. However, since the guideline for organic certification prohibits the use of any drugs in the absence of illness, few options are available on organic farms for the treatment of BRD. According to our survey results, although more than half of the farms administered vaccinations against the three major causes of BRD (i.e., infectious bovine rhinotracheitis, parainfluenza 3, and bovine respiratory syncytial virus), the perceived disease frequency remained high. Some efforts have recently been introduced to reduce the prevalence of BRD in organic cattle dairies with the implementation of a new risk assessment tool to identify and subsequently control farm-specific risk factors that can favor BRD in calves [48]. Additionally, several preventative measures are promising against BRD, such as the administration of salable milk [49]. 

With regards to preventive measures, most of the participating farms reported having routine veterinarian visits (88.2%; Table S1), which was higher than organic farms in NY, WI, and OR (36%) [12]. This may be linked to the larger size of organic farms in CA, as studies have previously found a positive association between the frequency of routine veterinarian visits and the size of organic farms [22,50]. Vaccination rates against frequent diseases (i.e., *E. coli* mastitis, BVD) or zoonoses (i.e., leptospirosis, brucellosis) were as high as 81–92% in this study, when only 64–67% of the organic farms in NY, WI, and OR, reported using vaccines [12]. This difference may be associated with the frequency of routine veterinarian visits, and the establishment of herd health and preventive disease protocols (i.e., vaccinations, best husbandry practices). 

Regarding the use of medications or supplements, more organic farms reported using vitamin A-D-E and selenium in MN than in CA (74.3% versus 54% and 65.7% versus 54%, respectively), whereas organic farms in CA were more likely to limit potassium in dry cows (50%) for the prevention of hypocalcemia in fresh cows, than in MN (22.9%) [4]. As for antibiotics, which lead to the subsequent loss of organic status, their most common uses were treating respiratory diseases (47.0%) and mastitis (29.4%). Similarly, respiratory disease was the most common reason for using antibiotics in cows in organic dairies in MI, MN, NY, and WI [13]. However, this phenomenon contrasted with the results from a recent survey in CA and from a study in other states, in which none of the organic dairies reported using antibiotics to treat mastitis [13,15]. There are currently limited options for alternative treatments to antibiotics with proven efficacies, and there is a need to investigate these efficacies through clinical trials [25,29,33]. This is all the more important as farmers may delay antimicrobial treatment, opting for alternative therapies to retain organic status, potentially leading to animal welfare issues —a concern that was previously expressed by organic dairy cattle farmers interviewed in OH [25]. 

Interestingly, regarding parasite specific issues, most of the farm owners did not perceive gastrointestinal parasites or biting flies to be a problem and did not identify signs of lice or mites. Many, therefore, did not consider internal parasite control when scheduling grazing (61.8%; Table S2). These results are similar to those of a parasite management study in organic dairy farms in MN, in which only 20% of producers perceived gastrointestinal parasites as a problem [14]. However, controlling flies was reported as the biggest challenge (89%) for organic dairy farmers in a Northeast US study [51]. Half of the organic dairy producers interviewed in OH also stated that fly control was one of their biggest challenges [25]. Ivermectin was added to the list of prohibited substances for livestock in the NOP in January 2019 [52], but an initial proposal to remove ivermectin from the NOP written by the National Organic Standard Board stated that ivermectin was the preferred parasiticide in Western states of the US [53]. Since ivermectin was prohibited during our survey period, the potential impacts of its prohibition on parasite problems could not be assessed.

Our study had several limitations. Participation in this survey was entirely voluntary, which may have induced a lack of representativeness. Even with the mixed-mode approach, and the use of a financial incentive, our overall response rate was 22.5% (n = 36), which is relatively low, but higher than in other CA studies conducted in 2018 (15.1%, n = 16) [16] and in 2019 (n = 18) [15]. Northern CA had the highest response rate (20.8%), followed by Northern San Joaquin Valley (10.0%) and Greater Southern CA (0.0%). Potential bias due to regional differences is, however, unlikely as 90.0% (144/160) of organic dairy farms are located in Northern CA. Only a few participants (5.6–55.6%) indicated the perceived general efficacy of the various medications or supplements, which prevented us from presenting and interpreting results for these questions (Table S3). In addition, their efficiency was not assessed for specific diseases/disorders. We did not ask respondents to detail the use of vaccines or the types of housing by age class, which would have allowed us to investigate associations with disease/disorders. Specifically, pinkeye was the most frequent disease in heifers, but we did not include the vaccine for pinkeye in the list of vaccines being used. Future studies in CA should focus on comparing organic and conventional dairy farms, as has been done in other states. They should also examine the various challenges encountered by organic dairy farmers (i.e., organic certification requirements, animal nutrition, organic treatment options), so that areas of action could be prioritized.

## 5. Conclusions

This study described demographics, management practices, and the farmers’ perceptions of animal diseases on organic dairy cattle farms in CA. The results contribute to comparing the characteristics of organic dairy cattle farms in CA with those in other states in the US reported by previous research. Additionally, our results show that organic dairy cattle farms commonly reported diseases in all age classes despite high vaccination rates. They also highlight the importance of further exploring options and efficacies of alternative therapies through clinical trials, which currently remain limited. Efficacies of other preventive management practices should also be thoroughly examined. 

## Figures and Tables

**Table 1:** Demographics of organic dairy farms in CA (n = 36 farms surveyed).

Farm-Level Characteristics	Answers	Counts	Percentage (95%CI)
Predominant breed (>50%) (n = 35)	Holstein	21	60.0% (43.6–74.4%)
	Jersey	11	31.4% (18.6–48.0%)
	Crossbreed	3	8.6% (3.0–22.4%)
Seasonal calving (n = 36)	Yes	3	8.3% (2.9–21.8%)
	No	33	91.7% (78.2–97.1%)
New animals brought into the operation in the past 12 months (n = 34)	Yes	10	29.4% (16.8–46.2%)
	No	24	70.6% (53.8–83.2%)
Average bulk tank somatic cell counts (BTSCC, cells/mL) (n = 35)	Less than 100,000	3	8.6% (3.0–22.4%)
	100,000~199,000	24	68.6% (52.0–81.4%)
	200,000~299,000	8	22.9% (12.0–39.0%)
	More than 300,000	0	0%
Primary milk parlor (n = 36)	Herringbone or Parabone	17	47.2% (32.0–63.0%)
	Flat barn	11	30.6% (18.0–46.9%)
	Parallel (side-by-side)	5	13.9% (6.1–28.7%)
	Side-opening (tandem)	3	8.3% (2.9–21.8%)
Use of individual identification (n = 36)	Yes	35	97.2% (85.8–99.5%)
	No	1	2.8% (0.5–14.2%)
Use of record keeping program (n = 36)	Yes	34	94.4% (82.0–98.0%)
	No	2	5.6% (1.5–18.1%)

**Table 2:** Pasture and housing management (n = 35 farms ^1^).

Farm-Level Characteristics	Answers	Counts	Percentage (95%CI)
Grazing year-round (n = 35)	Yes	15	42.9% (28.0–59.1%)
	No	20	57.1% (40.9–72.0%)
Main method used for pasture management (n = 35)	Rotational grazing	20	57.1% (40.9–72.0%)
	Strip grazing	9	25.7% (14.2–42.1%)
	Mob grazing	5	14.3% (6.3–29.4%)
	All three	1	2.9% (0.5–14.5%)
Grazing on the same pasture with other livestock (n = 35)	Yes	3	8.6% (3.0–22.4%)
	No	32	91.4% (77.6–97.0%)
Housing type for lactating cows (n = 35)	Free stalls	25	71.4% (54.9–83.7%)
	Pastures	9	25.7% (14.2–42.1%)
	Bedded pack barn	1	2.9% (0.5–14.5%)
	Open lot/Dry lot	0	0%
Heat abatement method for lactating cows in summer (n = 14) ^2^	Shade (other than inside building)	9	64.3% (38.8–83.7%)
	Sprinklers or misters	4	28.6% (11.7–54.6%)
	Fans	3	21.4% (7.6–47.6%)
	None	3	21.4% (7.6–47.6%)

^1^ One of the 36 respondents did not answer questions in this part on pasture and housing management. ^2^ For this question, participants could select all that applied, and it was answered by 14 respondents.

## Data Availability

The de-identified data is available only for peer review as the dairy owners did not consent to publish it alongside the article. The IRB protocol requires that data collected be held confidential to prevent individual identification of a farm or business; as such, raw data from this study is not able to be shared.

## Supplementary Materials

The following supporting information can be downloaded at: https://www.mdpi.com/article/10.3390/ani12192526/s1, File S1: 2019 California Organic Dairy Survey Questionnaire; Table S1: health care of dairy animals in California organic farms; Table S2: parasite control and perception of organic dairy farms in California; Table S3: effectiveness of medications or supplements.
