# Supplementary material for: Survey of Management Practices and Farmers’ Perceptions of Diseases on Organic Dairy Cattle Farms in California
Source: Animals (Basel). 2022 Sep 21;12(19):2526. doi: 10.3390/ani12192526 (PMC9558946; doi:10.3390/ani12192526)
Supplement: Supplementary file 1 [file animals-12-02526-s001.zip › Supplemental File S1.pdf]

2019 CALIFORNIA ORGANIC DAIRY SURVEY

UC DAVIS VETERINARY MEDICINE   UC ANR University of California Agriculture and Natural Resources

PART 1: RESPONDENT INFORMATION

1.1) Please select the title that best describes **your position** at the organic dairy. *(Select one)*

❍ Herdsman/ Manager ❍ Employee (not manager)
❍ Owner ❍ Other (Specify): _______________

1.2) How **many years** have you been dairying? __________

1.3) Is your dairy farm/operation **currently certified organic**? ❍ YES ❍ NO

1.3.1) **IF YES,** what year did you receive your organic certification? __________

1.3.2) **IF YES,** what accredited certifying agent certified your dairy organic? *(Select one)*

❍ USDA ❍ Oregon Tilth Certified Organic ❍ Other (Specify): ______________
❍ CCOF ❍ Organic Certifiers, Inc.

1.4) What **county** is/are your farm(s) located within? ________________________

PART 2: FARM HISTORY AND DEMOGRAPHICS

2.1) What is the predominant **dairy breed** (>50% of the cows)? *(Select one)*

❍ Holstein ❍ Jersey
❍ Crossbreed ❍ Other (Specify): _______________

2.2) What is your **average milk production** per cow per day in gallons?__________ **gallons**

2.3) Which of the following best describes the **average BTSCC (Bulk Tank Somatic Cell Count)** for milk shipped during the last 12 months? *(Select one)*

❍ Less than 100,000 cells/ml ❍ 300,000 - 399,000 cells/ml ❍ 600,000 cells/ml or greater
❍ 100,000 - 199,000 cells/ml ❍ 400,000 - 399,000 cells/ml
❍ 200,000 - 299,000 cells/ml ❍ 500,000 - 599,000 cells/ml

2.4) What is your **primary milking parlor** used? *(Select one)*

❍ Side-opening (tandem) ❍ Rotary (Carousel) ❍ Other (Specify): __________________
❍ Herringbone (fishbone) ❍ Flat barn
❍ Parallel (side-by-side) ❍ Parabone (herringbone-parallel hybrid)

2.5) Do you use **individual identification** (ear tags, collars, electronic ID)? ❍ YES ❍ NO

2.6) Do you use any of the following type(s) of **record keeping program(s)**? ❍ YES ❍ NO

2.6.1) **If YES**, to you use the following systems? *(Check all that apply)*

**Record Keeping System**	
Handwritten records (e.g., a ledger or notebook)	☐
Dairy Herd Improvement Association (DHIA-Plus)	☐
DairyCOMP 305	☐
PCDart	☐
Other (Specify) ________________________	☐

2.7) Approximately how **many dairy animals** of each group (cows, heifers, calves) are currently on the farm?

Cows: ______ Heifers: ______ Calves: ______

2.8) Do you **calve seasonally**? ❍ YES ❍ NO

2.8.1) **If YES**, when? *(Select one)*

❍ Spring ❍ Spring & Fall
❍ Fall ❍ Other (Specify): ________________

2.9) In the past 12 months, were any cows, heifers, calves, or bulls **brought onto the operation**? ❍ YES ❍ NO

2.9.1) **If YES**, were they from *(Check all that apply)*:

**Source**	
Single Farm	☐
Multiple Farms	☐
Club Sale	☐
Dealer	☐
Sale Barn	☐
Other	☐

2.10) Approximately **how many** dairy animals (cows, heifers, calves (including bull calves)) were permanently removed for reasons other than death in last 12 months?

Cows: ______ Heifers: ______ Calves: ______

2.11) Please select **the frequency of where permanently removed or culled** dairy cows, heifers, and calves were sent in the last 12 months, excluding those that died.

**Cows**	**Never**	**Seldom**	**Often**	**Almost Always**
**Sent to another dairy**	❍	❍	❍	❍
**Sent to market, auction or stockyard**	❍	❍	❍	❍
**Sent directly to a packer or slaughter plant**	❍	❍	❍	❍
**Other (Specify): ____________________**	❍	❍	❍	❍

**Heifers**	**Never**	**Seldom**	**Often**	**Almost Always**
**Sent to another dairy**	❍	❍	❍	❍
**Sent to market, auction or stockyard**	❍	❍	❍	❍
**Sent directly to a packer of slaughter plant**	❍	❍	❍	❍
**Other (Specify): ____________________**	❍	❍	❍	❍

**Calves**	**Never**	**Seldom**	**Often**	**Almost Always**
**Sent to another dairy**	❍	❍	❍	❍
**Sent to market, auction or stockyard**	❍	❍	❍	❍
**Sent directly to a packer of slaughter plant**	❍	❍	❍	❍
**Other (Specify): _____________________**	❍	❍	❍	❍

2.12) Please select **the likelihood** of the **reasons why** dairy cows, heifers, and calves were **permanently removed or culled** in the last 12 months, excluding those that died.

Cows	Never	Unlikely	Likely	Very Likely
**Udder or mastitis** problems	❍	❍	❍	❍
**Lameness** or **injury**	❍	❍	❍	❍
**Reproductive** problems	❍	❍	❍	❍
**Low milk production** not related to above	❍	❍	❍	❍
Sold as **replacement** animals to another dairy	❍	❍	❍	❍
**Other** (Specify): _____________________	❍	❍	❍	❍

Heifers	Never	Unlikely	Likely	Very Likely
**Scours, diarrhea**, or other **digestive** problems	❍	❍	❍	❍
**Respiratory** problems	❍	❍	❍	❍
**Lameness** or **injury**	❍	❍	❍	❍
**Reproductive** problems	❍	❍	❍	❍
Sold as **replacement** animals to another dairy	❍	❍	❍	❍
**Other** (Specify): _____________________	❍	❍	❍	❍

Calves	Never	Unlikely	Likely	Very Likely
**Scours, diarrhea,** or other **digestive** problems	❍	❍	❍	❍
**Respiratory** problems	❍	❍	❍	❍
**Naval or joint** problems	❍	❍	❍	❍
Sold as **replacement** animals to another dairy	❍	❍	❍	❍
**Other** (Specify): _____________________	❍	❍	❍	❍

2.13) Approximately, **how many** dairy animals of each age group (cows, heifers, calves) **died or were euthanized**, in the last 12 months?

Cows: ______ Heifers: ______ Calves: ______

2.14) Please **select the frequency** of the **reasons** recognized **as the main cause** for **the death or euthanasia** of dairy animals in the past 12 months.

Cows	Never	Seldom	Often	Almost Always
**Digestive** (diarrhea, bloat, displaced abomasum)	❍	❍	❍	❍
**Respiratory** problems	❍	❍	❍	❍
**Metabolic** (hypocalcemia, ketosis)	❍	❍	❍	❍
**Mastitis or udder** problems	❍	❍	❍	❍
**Reproductive** (retained placenta, metritis)	❍	❍	❍	❍
**Lameness** or **Injury**	❍	❍	❍	❍
**Other** (Specify): _____________________________	❍	❍	❍	❍

(Question continued on the next page)

(Continued from the previous page)

2.14) Please **select the frequency** of the **reasons** recognized **as the main cause** for **the death or euthanasia** of dairy animals in the past 12 months.

**Heifers**	**Never**	**Seldom**	**Often**	**Almost Always**
**Digestive** (diarrhea, bloat)	❍	❍	❍	❍
**Respiratory** problems	❍	❍	❍	❍
**Reproductive**	❍	❍	❍	❍
**Lameness** or **Injury**	❍	❍	❍	❍
**Other** (Specify): ______________________________	❍	❍	❍	❍

**Calves**	**Never**	**Seldom**	**Often**	**Almost Always**
**Digestive** (diarrhea, bloat)	❍	❍	❍	❍
**Respiratory** problems	❍	❍	❍	❍
**Infectious** (Naval or joint infection, fever)	❍	❍	❍	❍
**Other** (Specify): ______________________________	❍	❍	❍	❍

PART 3: ANIMAL HEALTH MANAGEMENT

3.1) Please **select the frequency** of the following **diseases and disorders** that most impacted your dairy animals (cows, heifers, calves) in the last 12 months.

**Cows**	**Never**	**Seldom**	**Often**	**Almost Always**
**Digestive** (diarrhea, bloat, displaced abomasum)	❍	❍	❍	❍
**Respiratory**	❍	❍	❍	❍
**Metabolic** (milk fever, grass tetany)	❍	❍	❍	❍
**Mastitis**	❍	❍	❍	❍
**Reproductive** (retained placenta, metritis)	❍	❍	❍	❍
**Lameness**	❍	❍	❍	❍
**Pink Eye**	❍	❍	❍	❍
**Other (Specify)** ____________________				

**Heifers**	**Never**	**Seldom**	**Often**	**Almost Always**
**Digestive** (diarrhea, bloat)	❍	❍	❍	❍
**Respiratory**	❍	❍	❍	❍
**Reproductive**	❍	❍	❍	❍
**Parasites**	❍	❍	❍	❍
**Pink Eye**	❍	❍	❍	❍
**Other (Specify)** ________________	❍	❍	❍	❍

**Calves**	**Never**	**Seldom**	**Often**	**Almost Always**
**Digestive** (diarrhea, bloat)	❍	❍	❍	❍
**Respiratory**	❍	❍	❍	❍
**Parasites**	❍	❍	❍	❍
**Pink Eye**	❍	❍	❍	❍
**Infectious** (naval infection, joint infection, fever)	❍	❍	❍	❍
**Other (Specify)** _________________	❍	❍	❍	❍

3.2) Does your farm/operation normally treat with the following **medications or supplements**? How effective or ineffective do you perceive they were?

	YES	NO	Very Ineffective	Ineffective	Neither Effective nor Ineffective	Effective	Very Effective
Pharmaceutical Dewormers	❍	❍	❍	❍	❍	❍	❍
Coccidiostats in feed	❍	❍	❍	❍	❍	❍	❍
Vitamins A-D-E in feed	❍	❍	❍	❍	❍	❍	❍
Vitamins A-D-E injection	❍	❍	❍	❍	❍	❍	❍
Selenium in feed	❍	❍	❍	❍	❍	❍	❍
Selenium injection	❍	❍	❍	❍	❍	❍	❍
Ionophores in feed (e.g. Rumensin)	❍	❍	❍	❍	❍	❍	❍
Probiotics	❍	❍	❍	❍	❍	❍	❍
Limited potassium (in dry cow ration)	❍	❍	❍	❍	❍	❍	❍
Iodine products	❍	❍	❍	❍	❍	❍	❍
Herbal products	❍	❍	❍	❍	❍	❍	❍
Homeopathy	❍	❍	❍	❍	❍	❍	❍
Essential Oils	❍	❍	❍	❍	❍	❍	❍
Acupuncture	❍	❍	❍	❍	❍	❍	❍
Aromatherapy	❍	❍	❍	❍	❍	❍	❍
Other (Specify): ______________________	❍	❍	❍	❍	❍	❍	❍

3.3) In past 12 months, did you **use antibiotics**? ❍ YES ❍ NO

3.3.1.) Please list the **reasons** (**diseases**), **treatment**, and **how the antibiotic was applied** (oral, subcutaneous, intravenous, intramuscular injection)

____

____

____

3.4) Do you **vaccinate** for any of the following diseases at the dairy? *(Check all that apply)*

**Vaccine**	
BVD (Bovine Viral Diarrhea)	☐
IBR (Infectious Bovine Rhinotracheitis)	☐
P13 (Parainfluenza Type 3)	☐
BRSV (Bovine Respiratory Syncytial Virus)	☐
Rotavirus/Coronavirus	☐
Haemophilus somnus	☐
Lepto (Leptospirosis)	☐
Salmonella	☐
E. coli mastitis	☐
Clostriduim, such as black leg or enterotoxemia	☐
Brucellosis (heifers only)	☐
Johne’s disease (Mycobacterium paratuberculosis) (heifers only)	☐
Neospora	☐
Rabies	☐
Other (Specify) _____________________________	☐

3.5) Does your farm utilize **routine veterinarian** visits? ❍ YES ❍ NO

3.5.1) **If YES,** approximately how often are veterinarian visits? *(Select one)*

❍ More than once a month ❍ Once every 1-3 months ❍ Once every 4-6 months
❍ Once every 7-9 months ❍ Once every 10-12 months ❍ Once every 12 + months

3.6) Does your **veterinarian** (or most recent veterinarian) **provide** the following services? *(Check all that apply)*

**Veterinarian Services**	
Treatments for Sick Cows	☐
Drug Prescriptions	☐
Preventive/Prophylactic Measures	☐
Reproductive Work	☐
Nutritional Advice	☐
Other (Specify) ________________	☐

3.7) Who most commonly **identifies** sick dairy cows? *(Select one)*

❍ Herd Manager ❍ Employee ❍ Other (Specify): _______________
❍ Veterinarian ❍ Owner

3.8) Who most commonly **treats** sick dairy cows? *(Select one)*

❍ Herd Manager ❍ Employee ❍ Other (Specify): _______________
❍ Veterinarian ❍ Owner

3.9) Does your farm have **written protocols** for disease identification and treatment? ❍ YES ❍ NO

3.9.1) **If YES**, which is your **primary** source of information for disease treatment and management options for your herd? *(Select all that apply)*

☐ Herd Manager ☐ University Extension
☐ Organic certifier ☐ Internet resources
☐ Organic Co-Op ☐ Veterinarian
☐Printed media (feed magazines, etc.) ☐ Other (Specify): ________________________

3.10) How are **treatments recorded** for cows that received a treatment? *(Select one)*

❍ Written ❍ Both written and computerized ❍ Other (Specify): ___________________
❍ Computerized ❍ Treatments are not recorded for each cow

PART 4: PARASITE SPECIFIC QUESTIONS

4.1) When organizing the **grazing schedule**, do you consider intestinal parasite prevention/control?

❍ YES ❍ NO

4.2) Do you perceive **gastrointestinal parasites** to be a problem within your herd? ❍ YES ❍ NO

4.2.1) **If YES,** please select the age group you perceive to be the most affected. *(Select one)*

❍ Pre-weaned calves ❍ Post-weaned calves
❍ Heifers ❍ Cows

4.3) Do you use **any dewormers**? ❍ YES ❍ NO

4.3.1) **If YES,** do you use any of the following **treatments**? *(Select all that apply)*

**Treatment**	
Diatomaceous earth	☐
Ivermectin	☐
Moxidectin	☐
Fenbendazole	☐
Other (Specify) ______________________	☐

4.4) Do you have any animals showing signs of lice or mites, such as excessive scratching and rubbing, hair loss or scabby skin conditions, in particular around the tail or head? ❍ YES ❍ NO

4.4.1) **If YES,** do you use any of the following **treatments**? *(Select all that apply)*

**Treatment**	
Lice and mange wash	☐
Botanical insecticide (pyrethrum, garlic, essential oils)	☐
Mineral or dietary supplement	☐
Sulfur powder	☐
Emergency parasiticide	☐
Other (Specify) _______________________	☐

4.5) Do you perceive **biting flies** to be a problem within your herd? ❍ YES ❍ NO

PART 5: HOUSING AND PASTURE MANAGEMENT

5.1) Do you **graze** year-round? ❍ YES ❍ NO

5.2) Which of the following best describe the **housing type** for your lactating cows? *(Select one)*

❍ Free-stalls ❍ Bedded pack barn ❍ Other (Specify): _________________
❍ Pasture ❍ Open lot/Dry lot

5.3) Do you use the following **heat abatement methods** provided to lactating cows in the summer months? *(Select all that apply)*

**Heat Abatement Methods**	
Sprinklers or misters	☐
Fans	☐
Shade (other than inside building)	☐
Other (Specify) _____________________	☐

5.4) How many **acres** of designated organic pasture do you have on your farm? ______________ ACRES

5.5) What is the main method used for **pasture management**? *(Select one)*

❍ Strip grazing (temporary fencing separating portions of one grazing pasture for active and non-active grazing)
❍ Mob grazing (ultra-high livestock densities with a short grazing duration and a long rest period)
❍ Rotational grazing (moving grazing livestock between pastures)
❍ Other (Specify): _______________________________________

5.6) Do cows and heifers **graze** on the same pasture as **other livestock**? ❍ YES ❍ NO

5.6.1) **If YES,** what other animals graze with the cows? *(Select all that apply)*

☐ Poultry ☐ Pigs
☐ Goats ☐ Horses
☐ Sheep ☐ Other (Specify): _____________

2019 CALIFORNIA ORGANIC DAIRY SURVEY

PART 6: ORGANIC EDUCATION AND OUTREACH

6.1) What is your **preferred** method of receiving **information**? *(Select all that apply)*

☐ Reading (e.g., magazines, newsletters, fact sheets)
☐ Talking to people (e.g., farmers, farm advisors, veterinarians, company representatives, feed store employees)
☐ Internet
☐ Educational Meetings (e.g., workshops, seminars, webinars)
☐ Other (Specify): ________________________________

6.2) **Where** do you typically **obtain dairy health information**, such as disease prevention and treatment *(Select all that apply)*?

☐ Veterinarian ☐ UCCE Advisor
☐ Nutritionist ☐ Feed Store
☐ Sales representative ☐ Friends/Neighbors
☐ Internet ☐ Other (Specify): _______________

6.3) **How often** do you seek out this information?

❍ Never ❍ Daily ❍ Weekly ❍ Monthly ❍ Yearly

6.4) If you have any additional thoughts about the topics of the survey, the survey itself, or any additional questions that you feel would be helpful to ask please share them here.

____

____

____

2019 CALIFORNIA ORGANIC DAIRY SURVEY

********END OF THE SURVEY********

Thank you for participating in our survey.
Please mail this back in the self-addressed envelope that was provided.

To receive **your $20 participation gift card** please provide your information below. Your responses in the survey will not be linked to this information.

Name **(Optional)**: ____________________________ Dairy Name **(Optional)**: ______________________________

Street Address: ____

City: ______________________________________ Zip Code: _________________________________

Email **(Optional)**: ______________________

Please return to:
Alda Pires
