# Supplementary material for: Survey of Management Practices and Farmers’ Perceptions of Diseases on Organic Dairy Cattle Farms in California
Source: Animals (Basel). 2022 Sep 21;12(19):2526. doi: 10.3390/ani12192526 (PMC9558946; doi:10.3390/ani12192526)
Supplement: Supplementary file 1 [file animals-12-02526-s001.zip › Supplementary Table S1.pdf]

Table S1. Health care of dairy animals in California organic farms (n = 36 farms).

Farm-Level Characteristics	Answers	Counts	Percentage (95%CI)
Routine Veterinarian visit (n =34)	Yes	30	88.2% (73.4–95.3%)
	No	4	11.8% (4.8–26.6%)
Provided service from Veterinarian [1] (n =33)	Reproductive work	25	75.8% (59.0–87.2%)
	Treatment for sick cows	23	69.7% (52.7–82.6%)
	Drug prescriptions	21	63.6% (46.6–77.8%)
	Preventive/Prophylactic	13	39.4% (24.7–56.3%)
	Nutritional advice	6	18.2% (8.6–34.4%)
Person who commonly identifies sick dairy cows (n = 35)	Herd manager	17	48.6% (33.0–64.4%)
	Owner	10	28.6% (16.3–45.1%)
	Employee	8	22.9% (12.1–39.0%)
	Veterinarian	0	0%
Person who commonly treats sick cows (n = 35)	Herd manager	15	42.9% (28.0–59.1%)
	Owner	13	37.1% (23.2–53.7%)
	Employee	6	17.1% (8.1–32.7%)
	Veterinarian	1	2.9% (0.5–14.5%)
Use of written protocols for disease identification and (n = 34)	Yes	24	70.6% (53.8–83.2%)
	No	10	29.4% (16.8–46.2%)
How the treatment recorded for cows that received a treatment (n = 34)	Written	19	55.9% (39.5–71.1%)
	Both written and computerized	13	38.2% (23.9–55.0%)
	Computerized	1	2.9% (0.5–14.9%)
	Not recorded for each cow	1	2.9% (0.5–14.9%)

[1] This question could be selected all that applied and answered by 33 respondents.
