# Supplementary material for: Survey of Management Practices and Farmers’ Perceptions of Diseases on Organic Dairy Cattle Farms in California
Source: Animals (Basel). 2022 Sep 21;12(19):2526. doi: 10.3390/ani12192526 (PMC9558946; doi:10.3390/ani12192526)
Supplement: Supplementary file 1 [file animals-12-02526-s001.zip › Supplementary Table S2.pdf]

Table S2. Parasite control and perception of organic dairy farms in California (n = 36 farms).

Farm-Level Characteristics	Answers	Counts	Percentage (95%CI)
Consideration of intestinal parasite prevention/control when scheduling grazing (n = 34)	Yes<br>No	13<br>21	38.2% (23.9–55%)<br>61.8% (45–76.1%)
Perceiving gastrointestinal parasites to be a problem (n = 34)	Yes<br>No	11<br>23	32.4% (19.1–49.2%)<br>67.7% (50.8–80.9%)
Use of dewormers (n = 34)	Yes<br>No	13<br>21	38.2% (23.9–55%)<br>61.8% (45–76.1%)
Showing signs of lice or mites (n = 32)	Yes<br>No	8<br>24	25.0% (13.3–42.1%)<br>75.0% (57.9–86.7%)
Perceiving biting flies to be a problem (n = 32)	Yes<br>No	10<br>22	31.3% (18.0–48.6%)<br>68.8% (51.4–82.0%)
