# Supplementary material for: Survey of Management Practices and Farmers’ Perceptions of Diseases on Organic Dairy Cattle Farms in California
Source: Animals (Basel). 2022 Sep 21;12(19):2526. doi: 10.3390/ani12192526 (PMC9558946; doi:10.3390/ani12192526)
Supplement: Supplementary file 1 [file animals-12-02526-s001.zip › Supplementary Table S3.pdf]

Table S3. Effectiveness of medications or supplements (n = 36 farms).

Farm–Level Characteristics	Answers	Counts	Percentage (95%CI)
Pharmaceutical Dewormers<br>(n = 15)	Neither effective nor ineffective<br>Effective<br>Very effective<br>Not applicable	1<br>5<br>5<br>4	6.7% (1.2–3.0%)<br>33.3% (15.2–58.3%)<br>33.3% (15.2–58.3%)<br>26.7% (10.9–52.0%)
Coccidiostats in feed (n =6)	Effective<br>Very Effective<br>Not applicable	2<br>2<br>2	33.3% (9.7–70.0%)<br>33.3% (9.7–70.0%)<br>33.3% (9.7–70.0%)
Vitamin A-D-E in feed (n = 9)	Very ineffective<br>Neither effective nor ineffective<br>Effective<br>Not applicable	1<br>3<br>4<br>1	11.1% (2.0–43.5%)<br>33.3% (12.0–64.6%)<br>44.4% (18.9–73.3%)<br>11.1% (2.0–43.5%)
Vitamin A-D-E in injection<br>(n = 16)	Effective<br>Very effective<br>Not applicable	11<br>3<br>2	68.8% (44.4–85.4%)<br>18.8% (6.6–43.0%)<br>12.5% (3.5–36.0%)
Selenium in feed<br>(n = 13)	Very ineffective<br>Neither effective nor ineffective<br>Effective<br>Very effective<br>Not applicable	1<br>2<br>7<br>1<br>2	7.7% (1.4–33.3%)<br>15.4% (4.3–42.2%)<br>53.8% (29.1–76.8%)<br>7.7% (1.4–33.3%)<br>15.4% (4.3–42.2%)
Selenium in injection<br>(n = 11)	Effective<br>Very Effective<br>Not applicable	7<br>1<br>3	63.3% (35.4–84.8%)<br>9.1% (1.6-37.7%)<br>27.3% (9.7-56.6%)
Ionophores in feed<br>(n = 5)	Very Effective<br>Not applicable	3<br>2	60% (23.1–88.2%)<br>40% (11.8–76.9%)
Probiotics (n = 14)	Neither effective nor ineffective<br>Effective<br>Very effective<br>Not applicable	1<br>8<br>3<br>2	7.1% (1.3–31.5%)<br>57.1% (32.6–78.6%)<br>21.4% (7.6–47.6%)<br>14.3% (4.0–39.9%)
Limited potassium<br>(n = 16)	Effective<br>Very effective<br>Not applicable	9<br>6<br>1	56.3% (33.2–76.9%)<br>37.5% (18.5–61.4%)<br>6.3% (1.1–28.3%)
Iodine products<br>(n = 20)	Very ineffective<br>Effective<br>Very effective<br>Not applicable	1<br>9<br>8<br>2	5.0% (0.8–23.6%)<br>45.0% (25.8–65.8%)<br>40.0% (21.9–61.3%)<br>10.0% (2.8–30.1%)
Herbal products<br>(n = 16)	Very ineffective<br>Ineffective<br>Effective<br>Very effective<br>Not applicable	1<br>1<br>8<br>4<br>2	6.3% (1.1–28.3%)<br>6.3% (1.1–28.3%)<br>50% (28.0–72.0%)<br>25% (10.2–49.5%)<br>12.5% (3.5–36.0%)
Homeopathy (n = 7)	Neither effective nor ineffective<br>Effective<br>Not applicable	2<br>3<br>2	28.6% (8.2–64.1%)<br>42.9% (15.8–75.0%)<br>28.6% (8.2–64.1%)
Essential Oils (n = 7)	Neither effective nor ineffective<br>Effective<br>Very effective<br>Not applicable	1<br>1<br>3<br>2	14.3% (2.6–51.3%)<br>14.3% (2.6–51.3%)<br>42.9% (15.8–75%)<br>28.6% (8.2–64.1%)
